# The role of adenosine in alcohol-induced respiratory suppression

**DOI:** 10.1016/j.neuropharm.2022.109296

**Published:** 2022-10-29

**Authors:** Benton S. Purnell, Sydney Thompson, Tenise Bowman, Jayant Bhasin, Steven George, Brian Rust, Madhuvika Murugan, Denise Fedele, Detlev Boison

**Affiliations:** a Department of Neurosurgery, Robert Wood Johnson Medical School, Rutgers University, Piscataway, NJ, USA; b Brain Health Institute, Rutgers University, Piscataway, NJ, USA

**Keywords:** Adenosine, Alcohol, Breathing, Caffeine, Respiratory suppression

## Abstract

Alcohol-related poisoning is the foremost cause of death resulting from excessive acute alcohol consumption. Respiratory failure is crucial to the pathophysiology of fatal alcohol poisoning. Alcohol increases accumulation of extracellular adenosine. Adenosine suppresses breathing. The goal of this investigation was to test the hypothesis that adenosine signaling contributes to alcohol-induced respiratory suppression. In the first experiment, the breathing of mice was monitored following an injection of the non-selective adenosine receptor antagonist caffeine (40 mg/kg), alcohol (5 g/kg), or alcohol and caffeine combined. Caffeine reduced alcohol-induced respiratory suppression suggesting that adenosine contributes to the effects of alcohol on breathing. The second experiment utilized the same experimental design, but with the blood brain barrier impermeant non-selective adenosine receptor antagonist 8-sulfophenyltheophylline (8-SPT, 60 mg/kg) instead of caffeine. 8-SPT did not reduce alcohol-induced respiratory suppression suggesting that adenosine is contributing to alcohol-induced respiratory suppression in the central nervous system. The third and fourth experiments used the same experimental design as the first, but with the selective A_1_ receptor antagonist DPCPX (1 mg/kg) and the selective A_2A_ receptor antagonist istradefylline (3.3 mg/kg). Istradefylline, but not DPCPX, reduced alcohol-induced respiratory suppression indicating an A_2A_ receptor mediated effect. In the fifth experiment, alcohol-induced respiratory suppression was evaluated in *Adk*^+/−^ mice which have impaired adenosine metabolism. Alcohol-induced respiratory suppression was exacerbated in *Adk*^+/−^ mice. These findings indicate that adenosinergic signaling contributes to alcohol-induced respiratory suppression. Improving our understanding of how alcohol affects breathing may lead to better treatment strategies and better outcomes for patients with severe alcohol poisoning.

## 1. Introduction

Alcohol consumption contributes to more than 5% of global mortality (WHO, 2019). This problem is potentially becoming more serious given the increase in alcohol consumption and alcohol-related deaths during the COVID-19 pandemic (White et al., 2022). In the United States, alcohol-related poisonings are the primary cause of death attributable to the acute effects of alcohol with an incidence approximately twice that of alcohol-related motor vehicle accidents (CDC, 2022b).

Respiratory arrest is crucial to the pathophysiology of fatal alcohol poisoning (Lignian et al., 1983; Adinoff et al., 1988; Vale, 2007; Wang et al., 2019). Alcohol suppresses breathing under normal conditions (Wilson and Saukkonen, 2004; Langhan, 2013) and in response to hypoxia and hypercapnia (Sahn et al., 1975; Michiels et al., 1983; McCauley et al., 1988). Identifying the mechanisms of alcohol-induced respiratory suppression may lead to better treatment strategies for alcohol poisoning. Several hypotheses for alcohol-induced respiratory suppression have been put forward:

Acute alcohol consumption increases endogenous opioid signaling potentially via an increase in corticotrophin-releasing hormone (Gianoulakis, 2001; Lam et al., 2008). Opioid receptor activation suppresses breathing (Santiago and Edelman, 1985; Bachmutsky et al., 2020). Opioid receptor antagonism attenuates the effect of alcohol on respiratory responsiveness to hypoxia and hypercapnia in humans (Michiels et al., 1983). These observations have led to the hypothesis that endogenous opioid signaling contributes to alcohol-induced respiratory suppression (Michiels et al., 1983); however, this hypothesis is somewhat undermined by regional differences in alcohol-induced opioid release. Breathing is primarily controlled by brainstem structures (Smith et al., 1991; Del Negro et al., 2018). Alcohol at high doses increases opioid signaling in the forebrain, but not the brainstem (Lam et al., 2008; Jarjour et al., 2009). Furthermore, opioid receptor antagonism does not counteract alcohol-induced respiratory arrest in animal models (Lignian et al., 1983). These observations do not support a purely opioid based interpretation of alcohol-induced respiratory suppression (Lam et al., 2008; Jarjour et al., 2009).

Acute alcohol exposure increases GABAergic signaling via both presynaptic and postsynaptic mechanisms (Nestoros, 1980; Roberto et al., 2004; Weiner and Valenzuela, 2006). GABAergic signaling contributes to respiratory rhythmogenesis (Bonham, 1995), but excessive GABA receptor activation suppresses breathing (Hedner et al., 1981; Yamada et al., 1981). GABA receptor antagonism counteracts the respiratory effects of alcohol and barbiturate coadministration (Ren et al., 2012). Similarly, facilitation of glutamatergic signaling via AMPA receptors counteracts the respiratory effects of alcohol (Xiao et al., 2020). A case report indicates that GABA receptor antagonism may alleviate alcohol-induced respiratory suppression (Linowiecki et al., 1992); however, this effect has never been empirically investigated.

Alcohol affects the central nervous system through a variety of disparate mechanisms beyond endogenous opioids and GABA. Among those, alcohol causes extracellular adenosine levels to rise by two distinct mechanisms: (1) Alcohol is metabolized into acetaldehyde, and then acetate that, along with ATP, produces acetyl-CoA and AMP which is then converted into adenosine (Carmichael et al., 1991; Pardo et al., 2013). (2) Alcohol impairs adenosine reuptake through equilibrative nucleoside transporter 1 (Nagy et al., 1990). Adenosine is an inhibitory modulator of neuronal activity (Dunwiddie and Masino, 2001). Excessive adenosinergic signaling suppresses breathing under normal conditions (Eldridge et al., 1984, 1985; Lagercrantz et al., 1984) and in response to hypercapnia (Falquetto et al., 2018). Increases in extracellular adenosine are associated with the respiratory dysregulation seen in traumatic brain injury (Lusardi et al., 2020) and sudden unexpected death in epilepsy (Shen et al., 2010; Ashraf et al., 2021). Experimental manipulations in animal models have implicated increased adenosinergic signaling in the effect of alcohol on vigilance state (Dar et al., 1983; El Yacoubi, 2003; Fang et al., 2017), motor control (Dar, 1990; Connole et al., 2004), memory (Spinetta et al., 2008; Lopez-Cruz et al., 2016), portal vein blood flow to the liver (Orrego et al., 1988), and anxiety (Prediger et al., 2004). The goal of this investigation was to test the hypothesis that adenosine signaling contributes to the effects of alcohol on breathing. To test this hypothesis we quantified alcohol-induced respiratory suppression under conditions of: (1) systemic non-selective adenosine receptor antagonism; (2) peripheral non-selective adenosine receptor antagonism; (3) systemic A_1_ and (4) A_2A_ selective adenosine receptor antagonism; and (5) transgenic downregulation of adenosine metabolism. Clarification of the mechanistic underpinnings of alcohol-induced respiratory suppression may lead to the development of more effective treatment strategies and better outcomes for patients with severe acute alcohol poisoning.

## 2. Materials and methods

### Ethical approval

All procedures and protocols used in this study were approved by the Rutgers University Institutional Animal Care and Use Committee in accordance with the guidelines set by the Association for Assessment and Accreditation of Laboratory Animal Care and the National Research Council. Care was taken to use the minimum number of animals possible and to minimize their pain and distress.

### 2.1. Experimental design

#### 2.1.1. Experiment 1. determine whether systemic non-selective adenosine receptor antagonism reduces vulnerability to alcohol-induced respiratory suppression

Adult, male, C57BL6/J mice were injected with either caffeine (40 mg/kg, i.p.), alcohol (5 g/kg, i.p.), or a combination of alcohol (5 g/kg, i. p.) and caffeine (40 mg/kg, i.p.; *n* = 8–9 per group). The breathing of mice was monitored for 30 min before and 3 h after the injection. Each animal received one experimental trial of each condition in a counterbalanced order to avoid sequence effects. We predicted that animals treated with the non-selective adenosine receptor antagonist caffeine would undergo less severe alcohol-induced respiratory suppression than animals treated with alcohol alone, our data was consistent with this prediction.

#### 2.1.2. Experiment 2. determine whether peripheral non-selective adenosine receptor antagonism reduces vulnerability to alcohol-induced respiratory suppression

Adult, male, C57BL6/J mice were injected with either 8-(*p*-sulfo-phenyl)theophylline (8-SPT; 60 mg/kg, i.p.), alcohol (5 g/kg, i.p.), or a combination of alcohol (5 g/kg, i.p.) and 8-SPT (60 mg/kg, i.p.; *n* = 8–9 per group). 8-SPT is a non-selective adenosine receptor antagonist which, unlike caffeine, is unable to cross the blood brain barrier. The breathing of mice was monitored for 30 min before and 3 h after the injection. Each animal received one experimental trial of each condition in a counterbalanced order to avoid sequence effects. We predicted that animals peripherally treated with the non-selective adenosine receptor antagonist 8-SPT would undergo the same degree of alcohol-induced respiratory suppression as animals treated with alcohol alone, our data was consistent with this prediction.

#### 2.1.3. Experiment 3. determine whether systemic A_1_ receptor antagonism reduces vulnerability to alcohol-induced respiratory suppression

Adult, male, C57BL6/J mice were injected with either Dipropylcyclopentylxanthine (DPCPX; 1 mg/kg, i.p.), alcohol (5 g/kg, i.p.), or a combination of alcohol (5 g/kg, i.p.) and DPCPX (1 mg/kg, i.p.; *n* = 9 per group). The breathing of mice was monitored for 30 min before and 3 h after the injection. Each animal received one experimental trial of each condition in a counterbalanced order to avoid sequence effects. We predicted that animals treated with the A_1_ receptor antagonist DPCPX would undergo the same degree of alcohol-induced respiratory suppression as animals treated with alcohol alone, our data was consistent with this prediction.

#### 2.1.4. Experiment 4. determine whether systemic A_2A_ receptor antagonism reduces vulnerability to alcohol-induced respiratory suppression

Adult, male, C57BL6/J mice were injected with either istradefylline (3.3 mg/kg, i.p.), alcohol (5 g/kg, i.p.), or a combination of alcohol (5 g/kg, i.p.) and istradefylline (3.3 mg/kg, i.p.; *n* = 8–9 per group). The breathing of mice was monitored for 30 min before and 3 h after the injection. Each animal received one experimental trial of each condition in a counterbalanced order to avoid sequence effects. We predicted that animals treated with the A_2A_ receptor antagonist istradefylline would undergo less severe alcohol-induced respiratory suppression than animals treated with alcohol alone, our data was consistent with this prediction.

#### 2.1.5. Experiment 5. determine whether transgenic downregulation of adenosine metabolism increases vulnerability to alcohol-induced respiratory suppression

*Adk*^+/−^ mice are heterozygous for a genetic knockout of the adenosine kinase (*Adk)* gene which encodes the major metabolic (adenosine removing) enzyme ADK (Boison et al., 2002). Adult, male and female, *Adk*^+/+^ and *Adk*^+/−^ mice were injected with alcohol (5 g/kg, i.p.; *n* = 8–9 per group). The breathing of mice was monitored for 30 min before and 3 h after the injection. We predicted that animals with impaired metabolic adenosine clearance (*Adk*^+/−^) would experience more severe alcohol-induced respiratory suppression than animals with normal adenosine clearance (*Adk*^+/+^), our data was consistent with this prediction.

### 2.2. Animals and drug injections

The C57BL/6J mice used in this study were acquired from Jackson Labs at 7–8 weeks old (000664; Bar Harbor, ME). The *Adk*^+/−^ mice used in this study were produced, genotyped, and maintained as previously described (Boison et al., 2002). All mice were housed under normal 12:12 LD conditions (lights on at 0600, lights off at 1800). Mice had ad libitum access to food (PicoLab 5058; LabDiet, St. Louis, MO) and water. Following the end of experimentation, animals used in this study were euthanized by CO_2_ exposure followed by cervical dislocation.

All injections were given intraperitoneally. Injections of ethyl alcohol (Decon Labs; King of Prussia, PA), caffeine (Sigma-Aldrich; St. Louis, MO) and 8-SPT (United States Biological; Salem, MA) were given with 0.9% saline (Hospira; Lake Forest, IL) as a vehicle. Injections of DPCPX (Tocris Bioscience; Bristol, UK) and istradefylline (Sigma-Aldrich) were given with 20% dimethyl sulfoxide (DMSO; VWR International, Radnor, PA) and 80% saline as a vehicle. Alcohol injections were prepared at 20% v/v. An alcohol dose of 5 g/kg was selected with the expectation that it would generate substantive respiratory disruption without approaching the LD50 (Dinh and Gailis, 1979). Non-alcohol drug injections were prepared to have a volume of ~0.15 mL. In alcohol only, caffeine only, 8-SPT only, DPCPX only, and istradefylline only experimental trials, a saline or 20% DMSO injection of appropriate volume was also given so that animals in all three conditions in these experiments received approximately the same volume of injected fluid. Experimental trials were conducted at least 72 h apart to allow the animals to fully recover.

### 2.3. Whole body plethysmography

Mice were acclimated to the plethysmography chamber during two separate sessions of at least 30 min prior to experimentation. During experimental trials, animals were removed from their home cage, placed in the plethysmography chamber (Data Sciences International, St. Paul, MN) and breathing was recorded for 1 h. Data from the first 30 min of the initial recording period was not analyzed, data from the second 30 min was analyzed as pre-injection baseline. Following the baseline recording period, mice were removed from the plethysmography chamber, injected with alcohol and/or an adenosinergic drug, and returned to the chamber. Room air was drawn through the plethysmography chamber at 0.5 L/min. A barometric flow-through method (Drorbaugh and Fenn, 1955) was used to determine respiratory frequency, tidal volume, and minute ventilation. Minute ventilation was used to determine degree of respiratory suppression as this parameter most directly relates to the capacity of the animal to prevent blood gas derangement and the adverse health outcomes that might result; however, the other parameters have been included as they can provide crucial information regarding the cause of differences in overall ventilation. Stable, artifact free segments of breathing were identified and analyzed using a ‘Drorbaugh & Fenn Reduced Rejection’ algorithm with a 2 s log interval (FinePointe, Data Sciences International; Drorbaugh and Fenn, 1955).

### 2.4. Statistical analyses

Statistical analyses were conducted using GraphPad Prism 9 (GraphPad Software Inc.) or Microsoft Excel (Microsoft Corp.). Two-way ANOVAs on baseline-normalized time series data were used to evaluate differences between conditions. Unless otherwise specified, data are expressed as mean ± standard error. Group sizes were determined using power calculations based on the effect sizes seen in pilot data in the lab. Significance threshold was set at *p* < 0.05 for all comparisons.

## 3. Results

### 3.1. Systemic non-selective adenosine receptor antagonism reduces alcohol-induced respiratory suppression

To determine the effect of systemic non-selective adenosine receptor antagonism on alcohol-induced respiratory suppression, breathing was monitored in mice before and after an injection of caffeine, alcohol, or a combination of alcohol and caffeine.

**Fig. 1.**
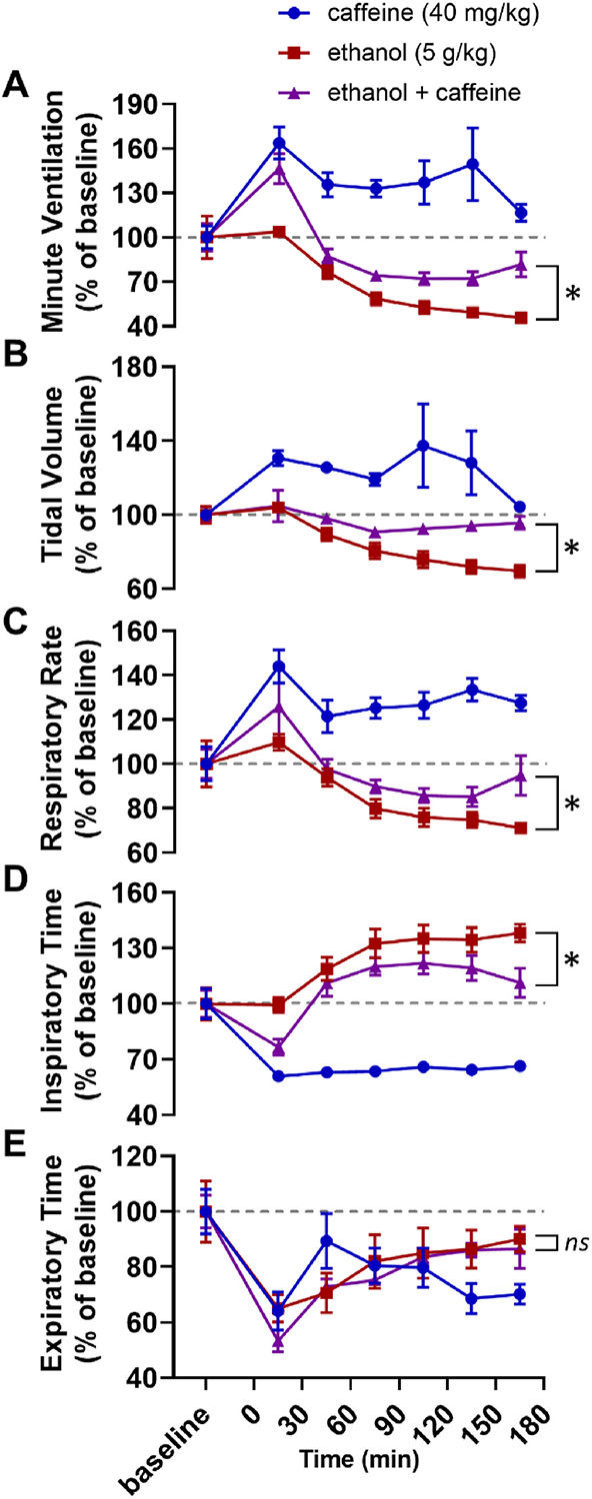
Caffeine reduces alcohol-induced respiratory suppression. Time series graphs depicting (A) minute ventilation, (B) tidal volume, (C) respiratory rate, (D) inspiratory time, and (E) expiratory time before and after injections of caffeine alone (40 mg/kg, blue circles), alcohol alone (5 g/kg, red squares), and alcohol with caffeine (5 g/kg alcohol and 40 mg/kg caffeine, purple triangles). *, *p* < 0.05; *ns*, *p* ≥ 0.05; two-way ANOVA. Data has been normalized to baseline and depicted as mean with SEM. Baseline is defined as the 30 min prior to drug administration. Grey dotted line, baseline level.

Two-way ANOVAs were used to compare baseline-normalized respiratory parameters between alcohol alone and caffeine with alcohol treatment conditions. Alcohol with caffeine experimental trials were significantly different than alcohol alone in minute ventilation (*F*_1,111_ = 34.98, *p* < 0.001; Fig. 1A), tidal volume (*F*_1,112_ = 31.43, *p* < 0.001.; Fig. 1B), respiratory rate (*F*_1,112_ = 8.04, *p* = 0.005; Fig. 1C), and inspiratory time (*F*_1,112_ = 15.93, *p* < 0.001; Fig. 1D), but not expiratory time (*F*_1,112_ = 0.83, *p* = 0.365; Fig. 1E).

### 3.2. Peripheral non-selective adenosine receptor antagonism does not reduce alcohol-induced respiratory suppression

To determine whether the effects of caffeine observed in Experiment 1 were due to adenosine receptor antagonism in the central nervous system or in the periphery, breathing was monitored in mice before and after an injection of 8-SPT, alcohol, or a combination of alcohol and 8-SPT. 8-SPT cannot cross the blood brain barrier; thus, its effects on adenosine receptors are limited to the periphery.

**Fig. 2.**
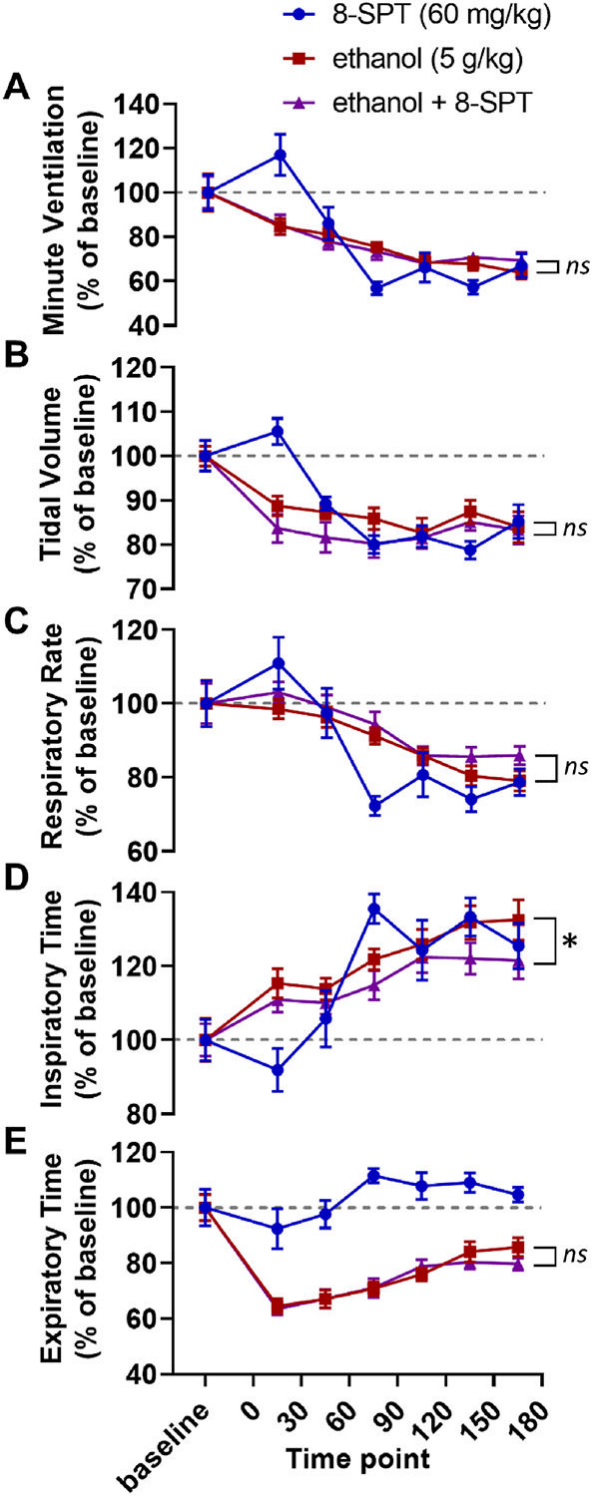
8-SPT does not reduce alcohol-induced respiratory suppression. Time series graphs depicting (A) minute ventilation, (B) tidal volume, (C) respiratory rate, (D) inspiratory time, and (E) expiratory time before and after injections of 8-SPT alone (60 mg/kg, blue circles), alcohol alone (5 g/kg, red squares), and alcohol with 8-SPT (5 g/kg alcohol and 60 mg/kg 8-SPT, purple triangles). *, *p* < 0.05; *ns*, *p* ≥ 0.05; two-way ANOVA. Data has been normalized to baseline and depicted as mean with SEM. Baseline is defined as the 30 min prior to drug administration. Grey dotted line, baseline level.

Two-way ANOVAs were used to compare baseline-normalized respiratory parameters between alcohol alone and 8-SPT with alcohol treatment conditions. There were no significant differences between alcohol alone and alcohol with caffeine in minute ventilation (*F*_1,105_ = 0.04, *p* = 0.843; Fig. 2A), tidal volume (*F*_1,105_ = 3.68, *p* = 0.058; Fig. 2B), respiratory rate (*F*_1,105_ = 3.15, *p* = 0.079; Fig. 2C) or expiratory time (*F*_1,105_ = 0.39, *p* = 0.533; Fig. 2D), but there was a difference in inspiratory time (*F*_1,105_ = 6.18, *p* = 0.015; Fig. 2C).

### 3.3. Systemic A_1_ receptor antagonism does not reduce alcohol-induced respiratory suppression

To determine whether the effects of adenosine receptor antagonism observed in Experiment 1 were mediated by the A_1_ receptor, breathing was monitored in mice before and after an injection of DPCPX, alcohol, or a combination of alcohol and DPCPX. Because DPCPX is selective to the A_1_ receptor, it should only counteract adenosinergic effects which are mediated by the A_1_ receptor.

**Fig. 3.**
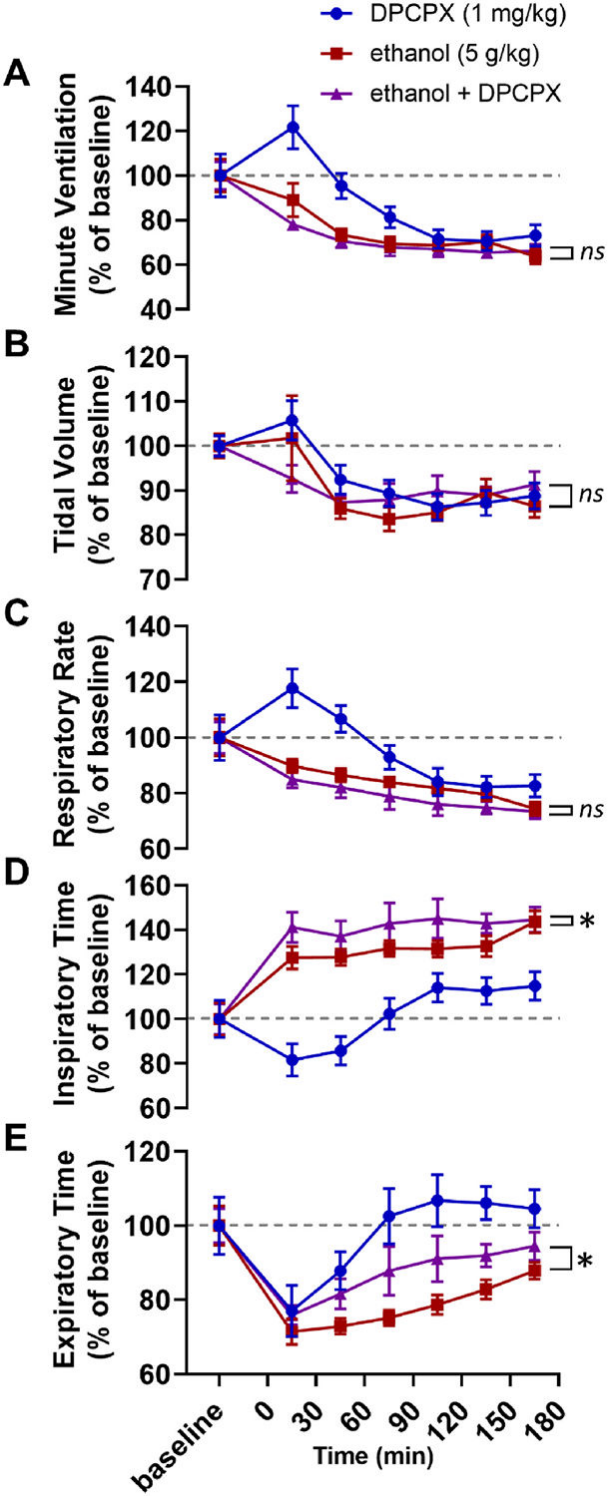
DPCPX does not reduce alcohol-induced respiratory suppression. Time series graphs depicting (A) minute ventilation, (B) tidal volume, (C) respiratory rate, (D) inspiratory time, and (E) expiratory time before and after injections of DPCPX alone (1 mg/kg, blue circles), alcohol alone (5 g/kg, red squares), and alcohol with DPCPX (5 g/kg alcohol and 1 mg/kg DPCPX, purple triangles). *, *p* < 0.05; *ns*, *p* ≥ 0.05; two-way ANOVA. Data has been normalized to baseline and depicted as mean with SEM. Baseline is defined as the 30 min prior to drug administration. Grey dotted line, baseline level.

Two-way ANOVAs were used to compare baseline-normalized respiratory parameters between alcohol alone and DPCPX with alcohol treatment conditions. Alcohol with DPCPX experimental trials were not significantly different than alcohol alone in minute ventilation (*F*_1,112_ = 1.54, *p* = 0.217; Fig. 3A), tidal volume (*F*_1,112_ = 0.16, *p* = 0.696; Fig. 3B), or respiratory rate (*F*_1,112_ = 3.68, *p* = 0.058; Fig. 3C). Alcohol with DPCPX experimental trials were significantly different than alcohol alone in inspiratory time (*F*_1,112_ = 6.54, *p* = 0.012; Fig. 3D) and expiratory time (*F*_1,112_ = 13.33, *p* < 0.001; Fig. 3E).

### 3.4. Systemic A_2A_ receptor antagonism reduces alcohol-induced respiratory suppression

To determine whether the effects of adenosine receptor antagonism observed in Experiment 1 were mediated by the A_2A_ receptor, breathing was monitored in mice before and after an injection of istradefylline, alcohol, or a combination of alcohol and istradefylline. Because istradefylline is selective to the A_2A_ receptor, it should only counteract adenosinergic effects which are mediated by the A_2A_ receptor.

**Fig. 4.**
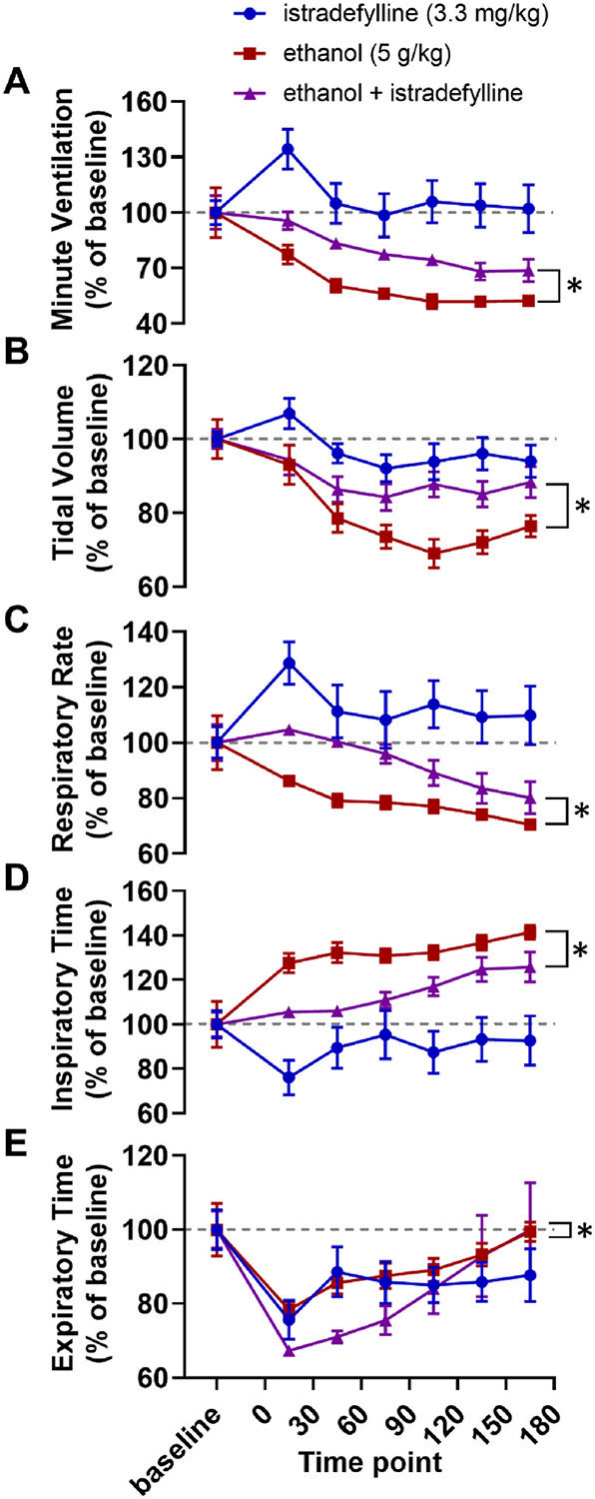
Istradefylline reduces alcohol-induced respiratory suppression. Time series graphs depicting (A) minute ventilation, (B) tidal volume, (C) respiratory rate, (D) inspiratory time, and (E) expiratory time before and after injections of istradefylline alone (3.3 mg/kg, blue circles), alcohol alone (5 g/kg, red squares), and alcohol with istradefylline (5 g/kg alcohol and 3.3 mg/kg istradefylline, purple triangles). *, *p* < 0.05; *ns*, *p* ≥ 0.05; two-way ANOVA. Data has been normalized to baseline and depicted as mean with SEM. Baseline is defined as the 30 min prior to drug administration. Grey dotted line, baseline level.

Two-way ANOVAs were used to compare baseline-normalized respiratory parameters between alcohol alone and istradefylline with alcohol treatment conditions. Alcohol with istradefylline experimental trials were different than alcohol alone in minute ventilation (F_1,105_ = 30.11, *p* < 0.001; Fig. 4A), tidal volume (F_1,105_ = 19.26, *p* < 0.001; Fig. 4B), respiratory rate (*F*_1,105_ = 29.71, *p* < 0.001; Fig. 4C), inspiratory time (*F*_1,105_ = 36.12, *p* < 0.001; Fig. 4D), and expiratory time (*F*_1,105_ = 4.13, *p* = 0.045; Fig. 4E).

### 3.5. Transgenic downregulation of adenosine metabolism increases alcohol-induced respiratory suppression

Adenosine kinase is the primary enzyme responsible for the metabolic clearance of adenosine (Boison and Jarvis, 2021). To determine whether transgenic downregulation of adenosine kinase would exacerbate alcohol-induced respiratory suppression, breathing was monitored in mice with impaired adenosine metabolism (*Adk*^+/−^) and their phenotypically normal littermates (*Adk*^+/+^) before and after an injection of alcohol.

**Fig. 5.**
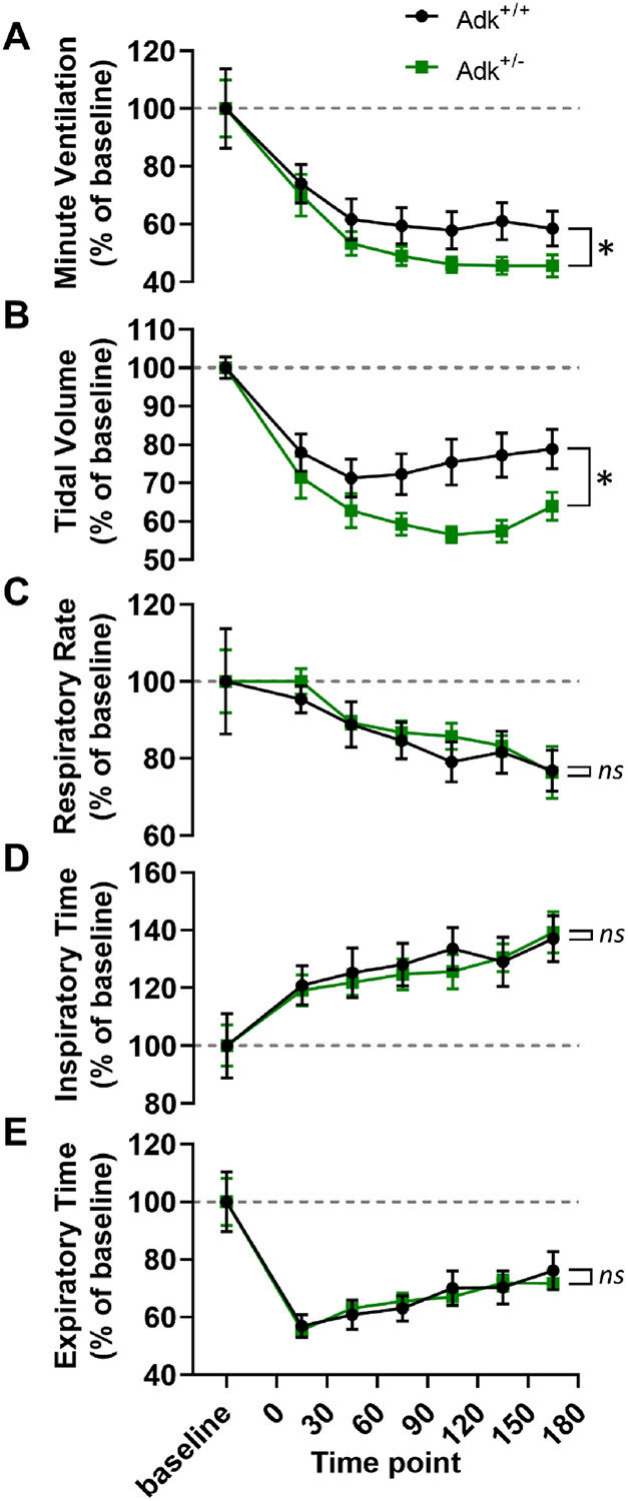
*Adk*^+/−^ mice are more vulnerable to alcohol-induced respiratory suppression. Time series graphs depicting (A) minute ventilation, (B) tidal volume, (C) respiratory rate, (D) inspiratory time, and (E) expiratory time before and after alcohol injection (5 g/kg) in adenosine kinase deficient (*Adk*^+/−^) and phenotypically wildtype (*Adk*^+/+^) mice. *, *p* < 0.05; *ns*, *p* ≥ 0.05; two-way ANOVA. Data has been normalized to baseline and depicted as mean with SEM. Baseline is defined as the 30 min prior to drug administration. Grey dotted line, baseline level.

Two-way ANOVAs were used to compare baseline-normalized respiratory parameters between *Adk*^+/−^ and *Adk*^+/+^ mice. Breathing following alcohol treatment was different between *Adk*^+/−^ and *Adk*^+/+^ mice in minute ventilation (*F*_1,105_ = 6.28, *p* = 0.014; Fig. 5A) and tidal volume (*F*_1,105_ = 25.30, *p* < 0.001; Fig. 5B), but not in respiratory rate (*F*_1,105_ = 0.47, *p* = 0.494; Fig. 5C), inspiratory time (*F*_1,105_ = 0.23, *p* = 0.634; Fig. 5D), or expiratory time (*F*_1,104_ = 0.02, *p* = 0.884; Fig. 5E).

## 4. Discussion

Alcohol generally, and acute alcohol poisoning specifically, are serious contributors to premature mortality (WHO, 2019; CDC, 2022b). The primary cause of mortality in acute alcohol poisoning is typically attributed to respiratory arrest (Lignian et al., 1983; Wang et al., 2019). The mechanistic antecedents of this respiratory dysfunction are not well understood. Given that (1) alcohol exposure is known to increase extracellular adenosine levels (Nagy et al., 1990), (2) excessive adenosine receptor activation suppresses breathing (Eldridge et al., 1984, 1985; Lagercrantz et al., 1984), and (3) adenosine has been implicated in other effects of alcohol (Dar, 1990; Prediger et al., 2004; Fang et al., 2017; Lusardi et al., 2020) we hypothesized that adenosine signaling contributes to the effect of alcohol on breathing. We tested this hypothesis, and further clarified adenosinergic contributions to alcohol-induced respiratory suppression, over the course of five experiments.

In the first experiment, a high dose of the non-selective adenosine receptor antagonist caffeine was administered with alcohol. We hypothesized that the effects of alcohol on breathing were mediated by adenosine. We expected that non-selective adenosine receptor blockade via caffeine would alleviate alcohol-induced respiratory suppression. Indeed, we observed that caffeine attenuated alcohol-induced reduction in ventilation suggesting that adenosine contributes to alcohol-induced respiratory suppression. (Fig. 1A). This effect was mediated by reduced suppression of both tidal volume (Fig. 1B) and, to a lesser extent, respiratory rate (Fig. 1C).

A critical detail in the interpretation of the data from the caffeine experiment (Exp. 1) is that breathing increased in response to caffeine alone (Fig. 1A). As a result, it is not possible to differentiate between (a) the action of caffeine on a mechanistic level (i.e. caffeine blocking adenosine receptors thereby counteracting the increased extracellular adenosine levels caused by alcohol) and (b) the action of caffeine as a stimulant which increases respiration (i.e. alcohol decreases breathing, caffeine increases breathing, giving caffeine with alcohol might increase breathing relative to alcohol alone without necessarily interacting with the underlying mechanism). Regardless, this limitation of the caffeine experiment was not present in the 8-SPT experiment (Exp. 2, Fig. 2A), the DPCPX experiment (Exp. 3, Fig. 3A), the istradefylline experiment (Exp. 4, Fig. 4A), or our transgenic approach (Exp. 5, Fig. 5A; the *Adk*^+/−^ and *Adk*^+/+^ mice had no differences in baseline breathing).

Metabolism and breathing are intimately linked processes (Wasserman, 1994) and it seems plausible that a drug that would increase breathing (e.g. caffeine) would increase metabolism in a way that would accelerate the clearance of alcohol from the blood. This increased alcohol metabolism due to caffeine might alleviate respiratory suppression without any direct involvement of adenosine signaling. The blood alcohol content of the mice used in this study was not measured as it would have necessitated removing the animal from the plethysmograph thereby disrupting the respiratory recording; however, this confound is unlikely given that it has been previously demonstrated that caffeine does not alter alcohol metabolism in humans (Azcona et al., 1995) or rodents (Kunin et al., 2000; Ferreira et al., 2004; Fritz et al., 2014). There is evidence to suggest that alcohol impairs caffeine metabolism (Mitchell et al., 1983), but this does not pose the same inter-pretational challenges for our results as the inverse.

In the second experiment, we evaluated whether the effects of adenosine receptor blockade observed in the first experiment were centrally or peripherally mediated. To test this, we peripherally administered the blood brain barrier impermeant non-selective adenosine receptor antagonist 8-SPT with alcohol. We hypothesized that the adenosinergic contributions to alcohol-induced respiratory suppression were occurring in the central nervous system (the rationale for this will be discussed in more detail below). As a result, we expected that peripheral non-selective adenosine receptor blockade via 8-SPT would not alleviate alcohol-induced ventilatory suppression. As predicted, 8-SPT did not reduce alcohol-induced respiratory suppression.

Alcohol increases adenosine signaling and affects breathing in both the brain and the periphery; however, its effects on breathing are regionally dichotomous. In the central nervous system adenosine and its analogs suppress breathing (Eldridge et al., 1984; Lagercrantz et al., 1984) and this suppression is counteracted by adenosine receptor antagonism (Eldridge et al., 1985). By contrast, in the periphery, adenosine analogs (Monteiro and Ribeiro, 1987) and inhibitors of adenosine metabolism (Monteiro and Ribeiro, 1989) stimulate breathing by increasing carotid body chemosensitivity. We hypothesized that adenosinergic contributions to alcohol-induced respiratory suppression are occurring in the central nervous system because if increased adenosine signaling in the periphery was the primary driving factor, it seems likely that alcohol would increase respiration, but the inverse is true (Wilson and Saukkonen, 2004; Langhan, 2013). In fact, increased adenosine signaling in the periphery might be having a beneficial effect on breathing which is outweighed by the detrimental effect in the central nervous system.

In the third and fourth experiments, we sought to determine the receptor responsible for adenosinergic contributions to alcohol-induced respiratory suppression. The results of Experiment 2 suggested that the effect of adenosine receptor antagonism on alcohol-induced respiratory suppression was occurring in the central nervous system. As a result, we focused on the A_1_ and A_2A_ receptors which are expressed in the brain (Dixon et al., 1996; Sheth et al., 2014). We hypothesized that the effects of alcohol on breathing were mediated by the A_2A_ receptor in light of its role in mediating other effects of alcohol, such as its hypnotic effects (El Yacoubi, 2003; Fang et al., 2017); however, given the effects of A_1_ receptor activation on breathing and chemoreception (Herlenius et al., 1997; Gettys et al., 2013; Falquetto et al., 2018) A_1_ receptor mediation of alcohol-induced respiratory suppression would not have been overly surprising. We expected that the A_2A_ receptor antagonist istradefylline, but not the A_1_ receptor antagonist DPCPX, would reduce alcohol-induced respiratory suppression. In accordance with our expectations, A_2A_ receptor antagonism, but not A_1_ receptor antagonism, reduced alcohol-induced ventilatory suppression (Figs. 3A and 4A). Like caffeine, the effect of istradefylline on ventilation was mediated by alleviated suppression of both tidal volume (Fig. 4B) and, to a lesser extent, respiratory rate (Fig. 4C).

In the fifth experiment, the effect of alcohol on breathing was evaluated in *Adk*^+/−^ mice which have an ~50% reduction in adenosine kinase (Boison et al., 2002). Adenosine kinase is the enzyme primarily responsible for adenosine metabolism (Boison and Jarvis, 2021). We hypothesized that adenosine kinase has a beneficial effect in the context of alcohol intoxication by facilitating the metabolic clearance of adenosine. As a result, we expected that *Adk*^+/−^ mice would undergo more severe alcohol-induced respiratory suppression. In accordance with our hypothesis, we found that alcohol-induced ventilatory suppression was exacerbated in *Adk*^+/−^ mice (Fig. 5A). As with the adenosine receptor antagonists, the effect on ventilation was primarily mediated by tidal volume (Fig. 5B) rather than respiratory rate (Fig. 5C).

The data collected using *Adk*^+/−^ mice does not suffer from the limitations of a pharmacological approach, but is subject to the caveats inherent to all constitutive transgenic manipulations. Reduced adenosine kinase expression might have resulted in compensatory changes during development that could have unpredictable second-order effects on physiology. Furthermore, this is an indirect manipulation of adenosine signaling that targets the enzymatic clearance of adenosine, not adenosine itself. Fortunately, the pharmacological and transgenic experimental approaches used in this investigation have nonoverlapping limitations and provide convergent evidence that adenosine contributes to alcohol-induced respiratory suppression.

The causal relationships between elevated adenosine signaling, the hypnotic effects of alcohol, and the respiratory suppression caused by alcohol are difficult to parse. Increased A_2A_ receptor activation contributes to the effect of alcohol on sleep (El Yacoubi, 2003; Fang et al., 2017), our data indicate that the same may be true of breathing. We hypothesize that increased A_2A_ receptor activation following alcohol exposure precipitates respiratory suppression and hypersomnolence by the same mechanism; however, on the basis of our results, we cannot rule out the possibility that alcohol increases A_2A_ receptor activation which causes hypersomnolence or frank sedation which then causes the respiratory effects. By the same count, previous studies indicating that adenosine contributes to other effects of alcohol exposure are not able to rule out the possibility that this is mediated by a change in breathing. In terms of correcting the respiratory suppression induced by alcohol in a clinical context, this distinction may not matter.

Currently, respiratory dysfunction consequent to severe alcohol poisoning is treated with intubation, artificial ventilation, supplemental oxygen and, in the most serious cases, hemodialysis (Adinoff et al., 1988; Vacca and Correllus, 2013; Atassi et al., 2018; Sauter et al., 2020). Mechanical ventilation is prone to serious side effects such as lung injury (Slutsky, 1999) and pneumonia (Chastre and Fagon, 2002). Pneumonia is of particular concern for individuals who are being treated for alcohol poisoning as alcohol use disorder nearly quadruples the risk of pneumonia becoming fatal (Lujan et al., 2010). Patient outcomes might be improved if an adenosine-based intervention could be used with, or instead of, existing approaches. Our results suggest that an intervention targeting adenosine signaling, perhaps with a high dose of caffeine, may counteract alcohol-induced respiratory suppression.

The United States Department of Health and Human Services, Centers for Disease Control and Prevention, and Federal Trade Commission caution against the concurrent consumption of caffeine and alcohol (FTC, 2010; DHHS, 2020; CDC, 2022a). Though our data suggest that caffeine counteracts alcohol-induced respiratory suppression, this investigation should not be viewed as casting doubt on the aforementioned guidance. The dangers of consuming caffeine and alcohol in large quantities are behaviorally mediated and occur prior to the point where alcohol might seriously affect breathing (Ferre and O’Brien, 2011; Emond et al., 2014). Specifically, by counteracting the sedative/hypnotic effects of alcohol, caffeine facilitates the consumption of additional alcohol potentially bringing the person closer to serious alcohol poisoning (Ferre and O’Brien, 2011). Furthermore, the dose of caffeine in this study was chosen as a tool to interrogate the mechanisms of alcohol-induced respiratory suppression, not to be analogous to recreational caffeine consumption. In an 80 kg human, an equivalent dose of caffeine (40 mg/kg) would be 3.2 g of caffeine or about 17 doses of coffee (McCusker et al., 2003). With that said, a lower dose might also have a greater effect. It has been observed in mice that caffeine at 25 mg/kg causes the righting reflex to be restored more quicky following alcohol exposure, but this effect is substantially reduced at 50 mg/kg and entirely absent at 100 mg/kg (El Yacoubi, 2003).

Future investigations should: (1) Evaluate the effect of concurrent reductions of adenosine, GABA, and endogenous opioid signaling on alcohol-induced respiratory suppression. (2) Determine whether the increase in adenosine receptor expression caused by chronic caffeine exposure increases vulnerability to alcohol-induced respiratory suppression (Fredholm, 1995). (3) Investigate whether adenosine signaling contributes to the dangerous interactions between alcohol and opiates (Hill et al., 2016) or benzodiazepines (Tanaka, 2002). (5) Lastly, given that traumatic brain injury elicits a surge in extracellular adenosine (Bell et al., 1998), it might be beneficial to explore the role of adenosine in the increased mortality seen in highly intoxicated traumatic brain injury patients with prior alcohol exposure (Tien et al., 2006).

## Data Availability

Data will be made available on request.

## Abbreviations:

ADK: adenosine kinase
GABA: γ-Aminobutyrie acid
